# Evaluation of clinical efficacy of silver-needle warm acupuncture in treating adults with acute low back pain due to lumbosacral disc herniation: study protocol for a randomized controlled trial

**DOI:** 10.1186/s13063-019-3566-2

**Published:** 2019-07-31

**Authors:** Tian Li, Siyao Wang, Shen Zhang, Xueyong Shen, Yanwen Song, Zhen Yang, Zouqin Huang

**Affiliations:** Shanghai Pudong New Area Hospital of Traditional Chinese Medicine, 399 Pingchuan Road, Pudong New Area, Shanghai, 201299 China

**Keywords:** Acupuncture therapy, Low back pain, Intervertebral disc degeneration, Radiculopathy

## Abstract

**Background:**

As a common cause of low back pain, lumbosacral disc herniation (LDH) is usually dealt with using non-surgical interventions. In the face of concerns about prescription opioid abuse, alternative and complementary treatments may be promising, among which silver-needle warm acupuncture is considered as an upgrading option for its potential anti-inflammatory and strong analgesic effect for patients with chronic pain. In this proposed study, we aim to assess its clinical efficacy in comparison with conventional stainless steel filiform-needle warm acupuncture.

**Methods/design:**

This is a randomized, two-armed, patient- and assessor-blinded trial. One hundred and sixty eligible patients recruited from December 2018 to June 2020 in three centers will be assigned for warm acupuncture treatment with either stainless steel filiform or silver needles. Nine sessions of 20-min treatment will be conducted during 3 consecutive weeks. Assessments with instruments including the Oswestry Disability Index, the visual analog scale, and the Japanese Orthopedic Association Back Pain Evaluation Questionnaire will be performed at four time points to explore the difference of clinical efficacy between two groups.

**Discussion:**

If the results show that participants treated with silver-needle warm acupuncture gain a greater improvement in terms of pain intensity, physical function, and quality of life, this study is expected to offer reliable evidence to widely push this treatment for LDH in clinical practice.

**Trial registration:**

Chinese Clinical Trial Registry, ChiCTR1800019051. Registered on 24 October 2018.

**Electronic supplementary material:**

The online version of this article (10.1186/s13063-019-3566-2) contains supplementary material, which is available to authorized users.

## Background and rationale

Lumbosacral disc herniation (LDH), usually referred as lumbosacral radiculopathy (LR) in Western countries [1], is one of the most commonly seen degenerative spinal disorders, accounting for 85% of low back pain with (or without) sciatica [2]. According to the “Chinese Consensus on Rehabilitation of Lumbar Disc Herniation,” first published in 2017, only 10–20% of LDH which might lead to severe neurologic deficits requires surgical intervention [3], indicating that the majority of patients with low back pain due to LDH can obtain symptomatic relief, though not a radical cure, simply through conservative therapies. However, the overuse of prescription opioids for analgesic purposes has turned out to be a great challenge characterized by substantial health care and societal costs [4]. Thus, public attention is gradually shifting to complementary and alternative medicine (CAM).

Among these alternatives under the CAM category, warm acupuncture, a combination of acupuncture therapy and thermal therapy (with a heat source like burning herbal moxa, laser light, or a multi-functional moxibustion apparatus), is often considered as an ideal option for patients who fail to benefit from manual or electrical acupuncture therapy. It is assumed that the steady and in-depth thermal conduction introduced by the inserted needle will exert a potent anti-inflammatory effect at the local lesions, leading to a strong analgesic effect [5].

Compared to the conventional stainless steel filiform type, needles made of precious metals (such as silver and gold) have been proven to have a better thermal effect in a warm acupuncture procedure, due to their higher thermal conductivity. The existing literature [6] suggests that, for a burning herbal moxa warm acupuncture procedure, a silver needle surpasses a stainless steel filiform needle, with a temperature of approximately 50 °C at the intradermal layer and approximately 40 °C at the tip. This temperature range is high enough to produce a positive effect on microcirculation at the local lesions without producing any discomfort or burns [7].

Taking the public concerns of opioid abuse and the favorable safety profile of acupuncture treatment into consideration, we have used the findings and experience from decades of clinical practice to design this randomized controlled trial (RCT), with the purpose of collecting reliable evidence for applying silver-needle warm acupuncture to low back pain caused by LDH.

### Objectives and hypothesis

In this RCT we intend to assess the clinical efficacy of silver-needle warm acupuncture versus stainless steel filiform-needle warm acupuncture on low back pain due to LDH by comparing the change of the Oswestry Disability Index (ODI), the visual analog scale (VAS), and the lumbar spine dysfunction score of the Japanese Orthopedic Association Back Pain Evaluation Questionnaire (JOABPEQ). We also expect to prove that silver-needle warm acupuncture can be an ideal conservative alternative for patients with acute low back pain who fail in conventional acupuncture therapy. Our hypothesis is that participants treated with silver-needle warm acupuncture will receive better improvement in severity of pain, physical function, and quality of life, both short and long term.

## Methods

### Study design and setting

**Fig. 1:**
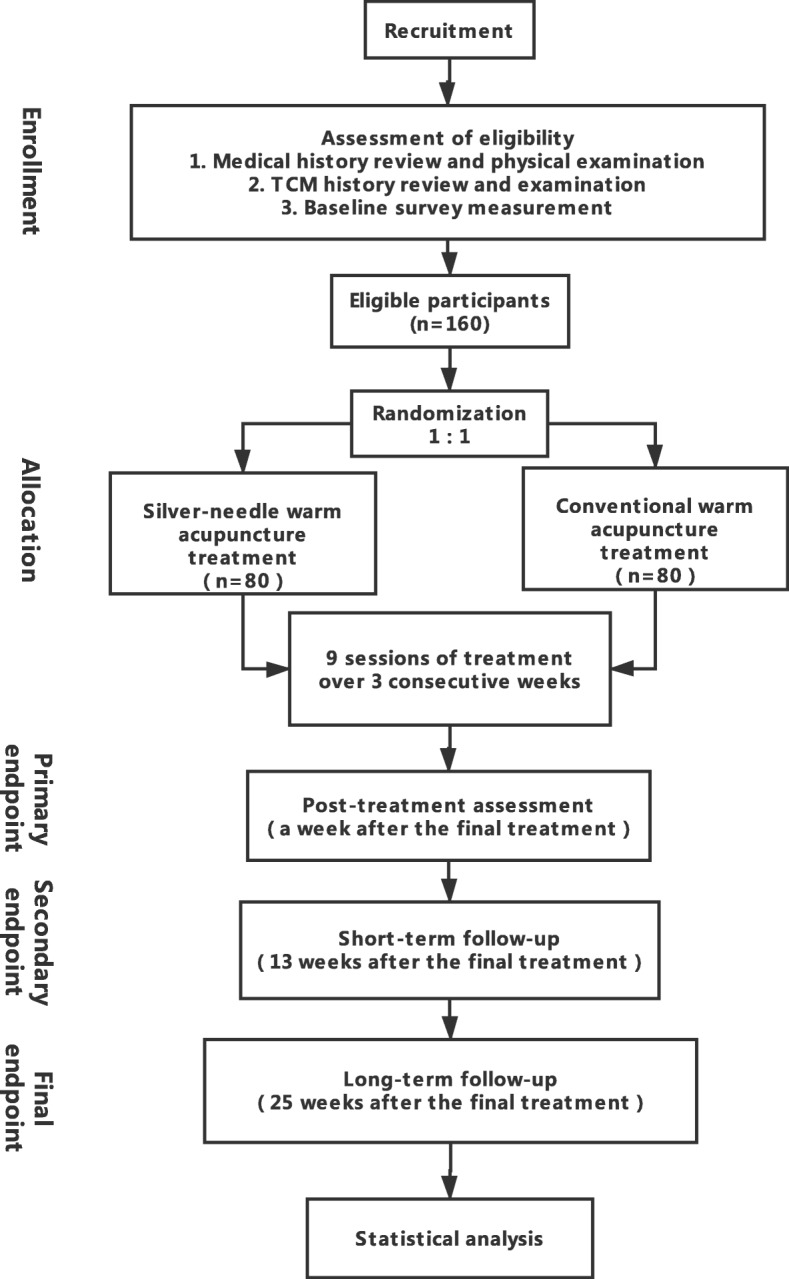
Study process: flowchart of study procedure

Developed according to the Standard Protocol Items: Recommendations for Interventional Trials (SPIRIT) Statement [8] (the SPIRIT checklist is shown in Additional file 1), this trial will be a randomized, two parallel-armed, patient- and assessor-blinded study conducted in three centers (Shanghai Pudong Hospital of Traditional Chinese Medicine (TCM), Longhua Hospital affiliated to Shanghai University of TCM, and Baoshan Hospital of Integrated Traditional Chinese and Western Medicine) in Shanghai from December 2018 to November 2021. Research results will be reported consistent with Consolidated Standards of Reporting Trials (CONSORT) guidelines [9] and the Revised Standards for Reporting Interventions in Clinical Trials of Acupuncture (STRICTA) [10]. A flowchart of this trial procedure is shown in Fig. 1. This RCT has been approved by the Ethics Committee of Shanghai Pudong Hospital of TCM on 11 October 2018, meaning all interested participants will sign a written informed consent form (see Additional file 4) with a clear understanding of the purpose, study procedure, and all potential risks of this trial. All written informed consent forms will be collected and obtained by the chief coordinator.

### Participant recruitment

Recruitment methods applied in this study include advertising through (1) social media (the official homepage or WeChat platform of Shanghai Pudong Hospital of TCM), (2) bulletin boards, and (3) flyers at affiliated community health service centers. Any potential participants can contact our researchers by our provided phone number and make an appointment for their screening visit. After potential participants have received a detailed explanation about the study protocol, only those who fully meet the selection criteria and sign the written informed consent form will be enrolled and randomly allocated to receive either silver-needle or stainless steel filiform-needle warm acupuncture treatment. The enrollment of participants was anticipated to start in December 2018 and end in June 2020.

### Sample size

The sample size calculation is conducted based on the result of a conventional therapy RCT [11], in which the post-treatment ODI score ($\overline{x}$ ± S) decreased to 26.29 ± 18.02 in the intervention group, and 34.58 ± 15.10 in the control group. It is indicated that, in the condition of α = 0.05 (two-sided) and 1 – β = 80%, the sample size of our proposed study should be 128 (64 for each group). Allowing for a sample loss of 20%, the final sample size is inflated to 160 in total with a 1:1 allocation ratio.

### Selection criteria

#### Inclusion criteria

We intend to include patients aged 20–60 who:

1. Have two of the following symptoms and signs confirmed by orthopedists of each center: (a) low back pain with or without lower extremity pain, lasting no more than 12 weeks and exacerbating with increased abdominal pressure; (b) scoliosis, paravertebral tenderness; (c) limited range of forward bending and a positive Lasegue’ s sign or Mackiewicz’ s sign; (d) paresthesia, dysreflexia, myodynamia, or even muscle atrophy in a lower extremity
2. Have imaging evidence (X-ray, computed tomography, or magnetic resonance imaging scan) supporting a confirmed diagnosis of LDH.
3. Indicate a VAS ≥ 5 on the first visit
4. Meet the criteria for the Cold-Dampness-Bi syndrome based on TCM theory determined by medical history review and examination [12] (cardinal symptoms such as recurrent ache, heaviness, or soreness in the lower back, cold limbs, restricted lumbar function; physical examination symptoms such as pale tongue with white or thick coating, sunken and tight pulse).

In the inclusion criteria, we emphasize the use of TCM diagnoses based on symptom differentiation for the following reasons. According to the theory of TCM, the Cold-Dampness syndrome is most commonly detected in patients with low back pain due to LDH. In this scenario, warm acupuncture is especially suitable for a Cold-Dampness syndrome pattern due to its function of dispelling cold, removing dampness, and warming and activating meridians. Also, acupuncturists should always tailor an acupoint prescription to be consistent with a patient’s real condition so that a better therapeutic effect can be achieved. However, this can be the source of performance bias. Thus, we want to target a community who can share a relatively standardized treatment protocol to ensure that the research finding is both rigorous and applicable.

#### Exclusion criteria

Participants will be excluded if they:

1. Suffer from cauda equina nerve damage, manifested as saddle anesthesia, bladder and bowel dysfunction, or sexual dysfunction; or suffer from LDH complicated by sciatica of the nerve trunk, piriformis syndrome, or other spinal diseases such as lumbar spinal stenosis, lumbar spondylolisthesis, ankylosing spondylitis, or spinal tuberculosis
2. Appear to have a life-threatening complication or contraindication to acupuncture therapy such as heart disease, liver or kidney dysfunction, poorly controlled diabetes mellitus, or coagulation dysfunction
3. Are pregnant or lactating women
4. Have had other therapy or treatment related to LDH (including kinesitherapy, manipulative therapy, traction therapy, physical therapy, pharmacotherapy such as non-steroidal anti-inflammatory drugs [NSAIDs], epidural injection of corticosteroids or anesthetics, or surgical therapy) in the past 3 months.

### Randomization and allocation concealment

An independent researcher (SW) will create a randomization sequence list using SPSS Statistics version 22.0 software and then transform it into a password-protected spreadsheet that contains the convert allocation schedule with an allocation ratio of 1:1. This spreadsheet will be run sequentially to generate a randomization number and treatment allocation for individual eligible participants after the screening visit by assessors (KZC, HMS). With the help of an allocation assistant, participants will only be informed of their own written randomization number, while project acupuncturists will be informed of both the randomization number and the corresponding treatment allocation through email.

### Blinding

Since this study is a procedure-related interventional RCT, the randomization charger, allocation assistant, and project acupuncturists are aware of the treatment allocation. Participants are blinded to their allocation because in both the intervention and control groups a prone position will be used during the treatment, and the difference of the needles and performance can hardly be detected by participants. The outcome assessors (KZC, HMS) and outcome statistician (TL) will also be blinded to the interventions received by the participants, because two of the three outcome measures are self-assessment instruments and are evaluated by the participants themselves.

### Trial procedure

#### Treatment providers

Four qualified acupuncturists (YWS, ZY, LPC, SZ), each with at least 10 years of practice experience, will conduct the treatment. They will have to finish a training session held by the chief coordinator (ZQH) that elaborates how to record the details of every single treatment on individual Case Report Forms (CRFs), how to conduct modified warm acupuncture with silver needles or conventional stainless steel needles, and obtaining their hands-on practical training.

#### Treatment rationale

Although acupuncture-related treatments, such as manual acupuncture, warm acupuncture, or electroacupuncture, have been applied for low back pain due to LDH for decades, and there are thousands of relevant clinical study reports, the most appropriate acupoint regimen has not yet been determined. In order to ensure that the study protocol is as rigorous as possible, we adopted the result of a literature review [13] and selected the most commonly used eight points (bilateral BL23, BL25, and BL54 and unilateral EX-B2 15/16 of the affected side; see Additional file 2) for low back pain due to LDH with Cold-Dampness syndrome as the core acupoints of a pragmatic regimen. This regimen allows the acupuncturist to add no more than a pair of other points based on the individualized tenderness, symptoms, or signs of each participant.

#### Treatment regimen

All participants will receive three sessions of 20-min treatment each week for 3 consecutive weeks, amounting to nine sessions in total.

The indoor temperature will be maintained at 25 °C by using a central air conditioner during treatment to minimize the influence of natural convection. In each session, the participant will be required to rest for 10 min in the treatment room before assuming a prone position.

After sterilizing the skin of selected points with alcohol pads (concentration of 75%), acupuncturists will insert the needles (0.40 mm * 75 mm) vertically to a depth of 30–50 mm and manipulate manually until the needle sensation (*Deqi*) is obtained. Needles on the eight core acupoints will be wrapped and fixed by the specially designed heat pipe, i.e., a tiny copper tube (2 mm * 0.5 mm * 20 mm) with a heatproof cork bottom, which will fit closely to the ceramic heat probe of a 15-output-set Multi-Function Moxibustion Apparatus (DAJ-23, XiangHe Chinese Traditional Medicine Instruments Co., Ltd., Heilongjiang, China; see Additional file 3). The output temperature of the device will be set in advance at 55 °C, implying that, after being attached to the ceramic heat probe for 2–3 min, the temperature at the intradermal part of the inserted needle will be stable and not exceed 50 °C. Twenty minutes later, acupuncturists will remove the ceramic heat probes, heat pipes, and needles cautiously and check thoroughly for any possible blisters or bleeding. During the trial, participants will be recommended to get more rest and avoid lifting of heavy loads or lumbar exertion (functional rehabilitation training like hip bridges, sit-ups, pull-ups, etc.) in their daily life.

The only difference between the two groups lies in the material of the needles. For the intervention group, we adopt the reusable, thin silver needles with a silver purity of 70 % (Hwato Medical Material Co., Ltd., Suzhou, China). To avoid contamination and cross-infection, every used silver needle will be strictly collected and stored in a special glass container (with a capacity of 20 needles) and then sent to the sterile department of each hospital for autoclave sterilization. An exclusive supervisor at each center will take charge of monitoring this procedure to ensure that only the silver needles with qualified disinfection can be reused. For the control group, the needles are disposable, stainless steel filiform needles (Jiajian Medical Material Co., Ltd., Wuxi, China) and will be applied for single use.

### Outcome measures

**Fig. 2:**
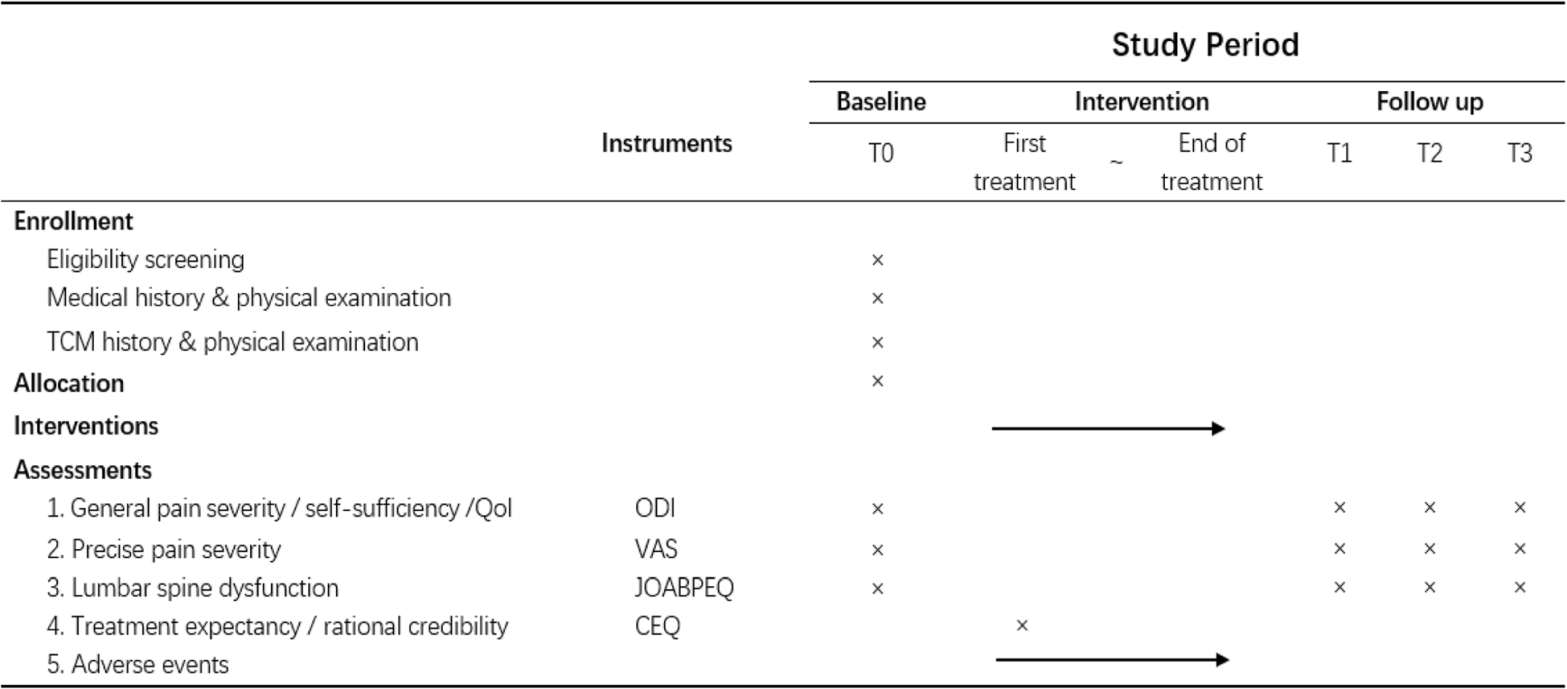
SPIRIT figure: schedule of enrollment, allocation, intervention, and assessments. *TCM* traditional Chinese medicine, *Qol* quality of life, *ODI* Oswestry Disability Index, *VAS* visual analog scale, *JOABPEQ* Japanese Orthopedic Association Back Pain Evaluation Questionnaire, *CEQ* credibility/expectancy questionnaire

The assessments consist of two self-assessment instruments and a physician-based questionnaire (all Chinese versions) in addition to a routine medical history review and physical examination. Assessments will be performed before allocation at screening (T0, baseline), a week after the final treatment (T1, primary endpoint), 13 weeks after the final treatment (T2, secondary endpoint), and 25 weeks after the final treatment (T3, final endpoint). The timeframe of data collection and assessments is shown in Fig. 2 (the SPIRIT figure).

#### Primary outcome measure

The primary outcome measure is the change in the ODI. The ODI involves 10 questions which mainly evaluate pain severity, self-sufficiency level, and quality of daily life [14]. Each question is calculated from 0 to 5 and then added up into an overall score. The higher the overall score is, the worse the general condition.

#### Secondary outcome measures

Secondary outcome measures include the following:

1. In order to determine the improvement of pain intensity more precisely, the visual analog scale (VAS) from 0 to 100 will be chosen as one of the secondary outcome assessment tools.
2. The Japanese Orthopedic Association Back Pain Evaluation Questionnaire (JOABPEQ) is a commonly used index in evaluating the treatment effect of LDH [15]. Considering its overlaps of the measurement of pain-related disorders, gait disturbance, social life disturbance, and psychological dysfunction with the ODI, we selectively assess three items in JOABPEQ which only evaluate the lumbar spine dysfunction.

In addition to these assessments, participants will also be required to complete an extra six-item-scale questionnaire [16] immediately after the first treatment, so that we can explore whether or not there is a significant difference in treatment expectancy and rationale credibility between the two groups.

#### Adverse events reporting and safety monitoring

Through the written informed consent form and detailed explanations, we ensure that every participant is aware of common adverse events related to acupuncture or warm needling treatment (such as bruising at the local area, sustained soreness or numbness, feeling faint, blistering due to overheating) as well as serious adverse events (classified as life-threatening, permanently incapacitating, or requiring hospitalization). During the treatment, participants must report any adverse events they experience to the project acupuncturists, while the latter must also record adverse events they themselves observe. All adverse events will be collected and presented promptly in detail to the assessors, who will decide on subsequent management (including intimate observation, additional medical management, or an early closure of participation) and record both the adverse events and the final outcome in the CRF.

### Data management and monitoring

A Data and Safety Monitoring Board (DSMB), mainly consisting of a statistics expert, an associate chief of osteology, and a general practitioner of TCM, will be set up prior to the first enrollment of participants. With unrestricted access to audit and investigate all individual participant data or interim results, the DSMB, totally independent from the sponsor and competing interests, will have the full right to make the final decision on the trial status.

The original data will be collected and recorded in the form of CRFs by the assessors, including a brief medical history, the evidence of LDH diagnosis, baseline information, treatment records, outcome of pre- and post-treatment assessments and follow-up assessments, adverse events, and compliance assessment. Any changes to these paper-based data forms will not be allowed without the investigation and authorization of the DSMB. Two faculty members (HX, QWZ) blinded to the allocation will take charge of the double entry of hard copy information into a custom-designed and password-protected database on the ResMan Research Manager of the Clinical Trial Management Public Platform.

### Statistical methods

In accordance with the intention-to-treat (ITT) principle, incomplete data will be collected, and reasons for missing data should be presented clearly so that the statistician can handle missing data with the appropriate statistical approach.

In general, all original data extracted from our online custom-designed database will be analyzed with SPSS Statistics version 22.0 software by a statistician. Continuous variables with a normal distribution will be expressed in the form of mean ± standard deviation, while continuous variables with a non-normal distribution or ordinal variables in the form of medians (with lower and upper quartiles), and categorical variables will be described as counts and proportions. To detect the statistically significant difference between the two groups, the independent sample *t* test will be performed for measurement data, the χ^2^ test for categorical data, and the Mann-Whitney *U* test for data that conform to an abnormal distribution. Any reported *p* value (two-sided) less than 0.05, with 95% confidence intervals, will be interpreted as the indication of a statistically significant difference.

## Discussion

In China, warm acupuncture is regarded as an upgraded acupuncture treatment for lumbosacral radiculopathy (LR) because of its combined therapeutic effect of both acupuncture and thermal therapy. It is assumed that the infrared rays introduced by the heat source can improve the blood circulation and enhance cell phagocytosis [17], leading to a strong anti-inflammatory effect around the responsible nerves [18], complete with the non-specific effect of acupuncture: the excitement of the endogenic opioid system [19].

According to thermology, the higher the temperature of an object is, the stronger the infrared rays it emits. Therefore, we assume that, for a warm acupuncture procedure, the thermal conducting property of the needle is essential, for it might influence the heat transfer and thus change the intensity of infrared rays so that, to some extent, it affects the clinical efficacy.

A study group [20] revealed that a silver needle, with seven times more heat transferred into the acupoints and the affected site, is superior to the most commonly applied stainless steel filiform type. This may be a potential explanation why, as reported in another recent study [21], silver-needle warm acupuncture could reduce the expression of neuronal nitric oxide synthase (nNOS) and substance P (SP) and increase the expression of 5-HT in the spinal cord [22], generating an analgesic effect [23].

An additional advantage in this proposed study is that we adopt an electrothermal moxibustion-imitating apparatus with a specially designed heat pipe to heat the needles. Compared to the conventional warm acupuncture procedure that uses burning moxa, which usually causes heavy smoke and burn injuries, the electrical heating device is feasible to avoid any unpleasant scent or air pollution. More importantly, according to the result of our pilot study, it can minimize the risk of burn injury by controlling the temperature at a relatively safe level. Thus, good patient compliance can be ensured.

### Trial status

Recruitment of participants started on 12 December 2018 and is anticipated to end on 30 June 2020. (The protocol is Version 1.0, dated 2018/12/1.)

## Additional files

Additional file 1: SPIRIT checklist. (DOC 122 kb)

Additional file 2: Locations of selected eight core points in the treatment regimen. (DOCX 600 kb)

Additional file 3: Silver-needle warm acupuncture conducted Multi-Function Moxibustion Apparatus with specially designed heat pipe. (DOCX 722 kb)

Additional file 4: Informed consent materials. (DOCX 17 kb)

## Data Availability

All data generated or analyzed in this study will be fully available without restriction through the online platform of the Chinese Clinical Trial Registry.

## Abbreviations

CAM: Complementary and alternative medicine
CEQ: Credibility/expectancy questionnaire
CRF: Case Report Form
DSMB: Data and Safety Monitoring Board
ITT: Intention-to-treat
JOABPEQ: Japanese Orthopedic Association Back Pain Evaluation Questionnaire
LDH: Lumbosacral disc herniation
LR: Lumbosacral radiculopathy
nNOS: Neuronal nitric oxide synthase
ODI: Oswestry Disability Index
Qol: Quality of life
RCT: Randomized controlled trial
SP: Substance P
SPIRIT: Standard Protocol Items: Recommendations for Interventional Trials
STRICTA: (Revised) Standards for Reporting Interventions in Clinical Trials of Acupuncture
TCM: Traditional Chinese medicine
VAS: Visual analog scale
