# Supplementary figures and images for: Evaluation of clinical efficacy of silver-needle warm acupuncture in treating adults with acute low back pain due to lumbosacral disc herniation: study protocol for a randomized controlled trial
Source: Trials. 2019 Jul 31;20:470. doi: 10.1186/s13063-019-3566-2 (PMC6668190; doi:10.1186/s13063-019-3566-2)

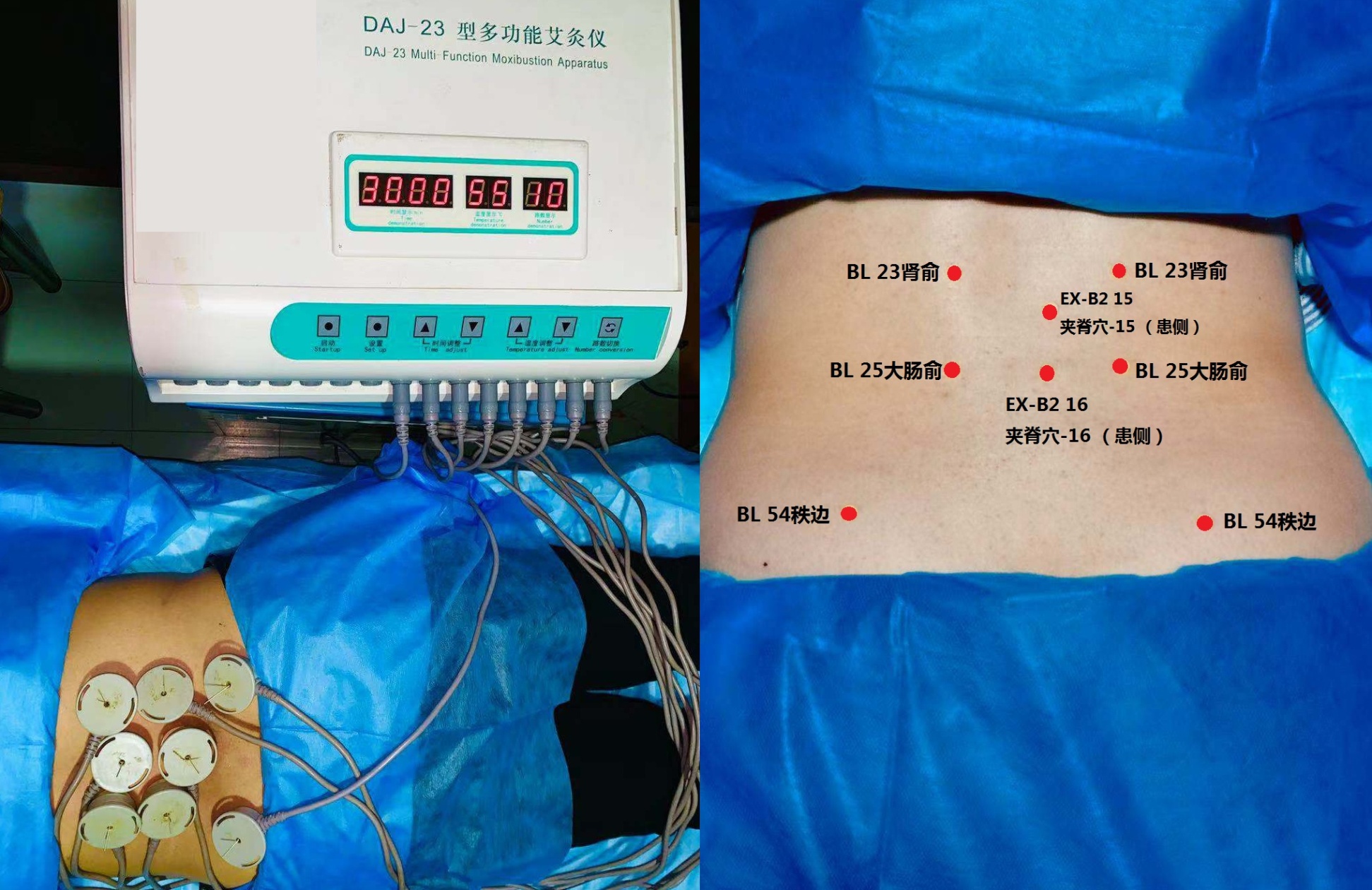

Supplement: Supplementary file 2 — Locations of selected eight core points in the treatment regimen. (DOCX 600 kb) [file 13063_2019_3566_MOESM2_ESM.docx]

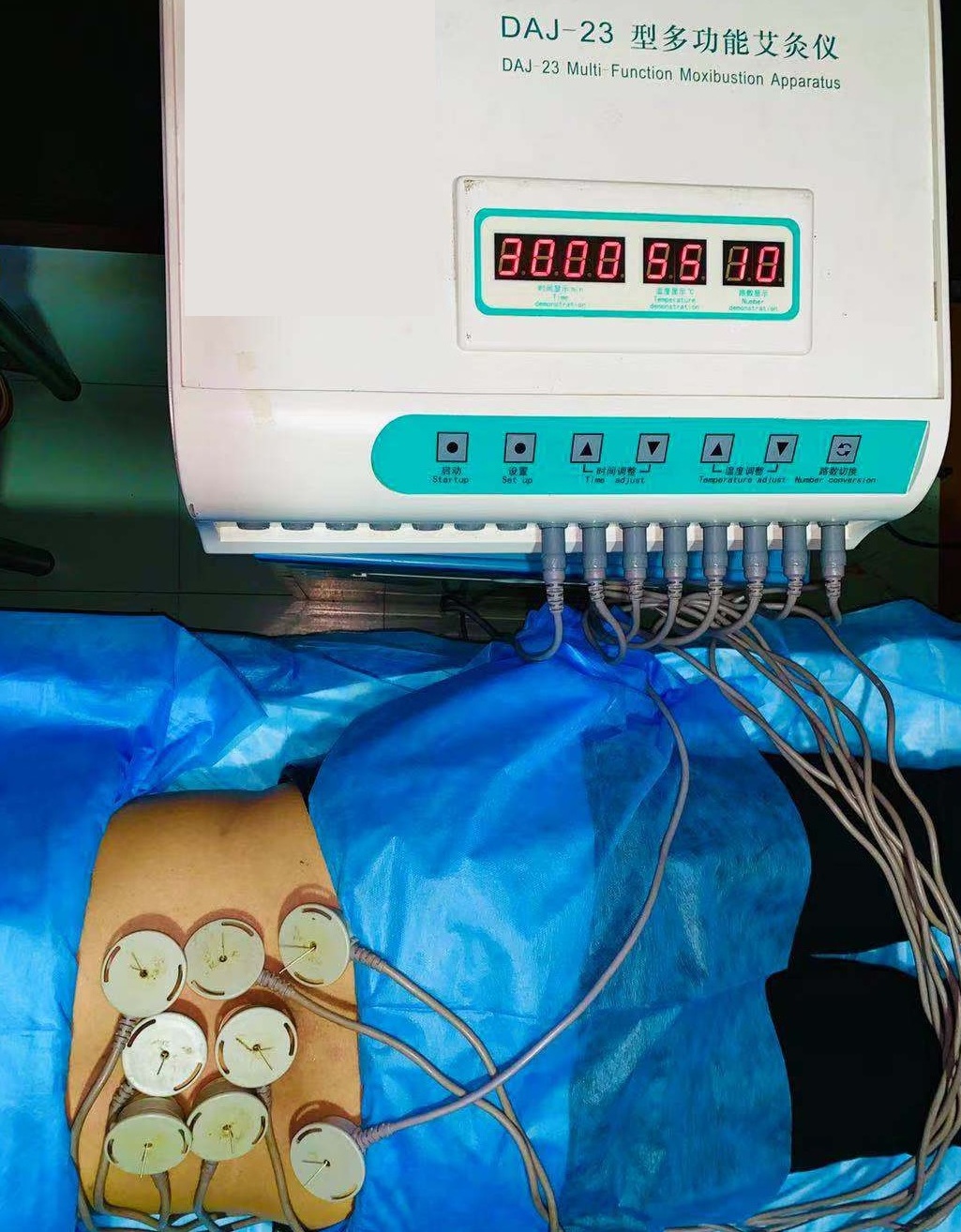


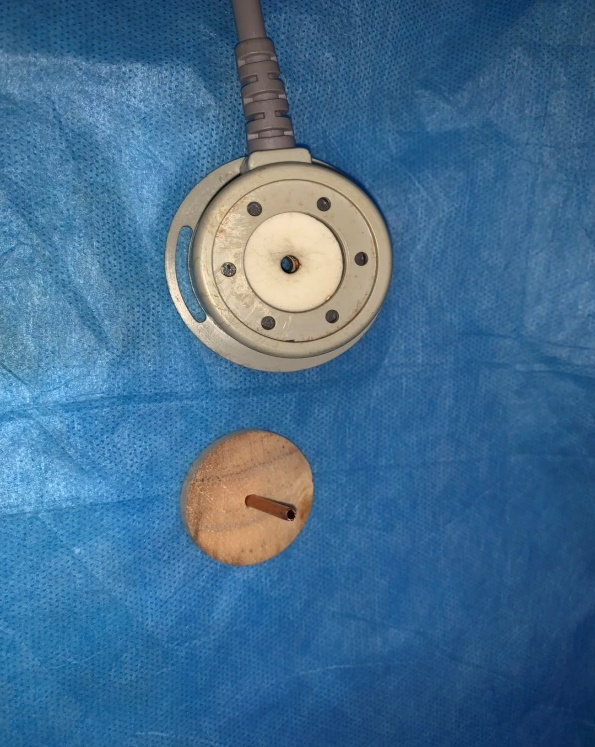


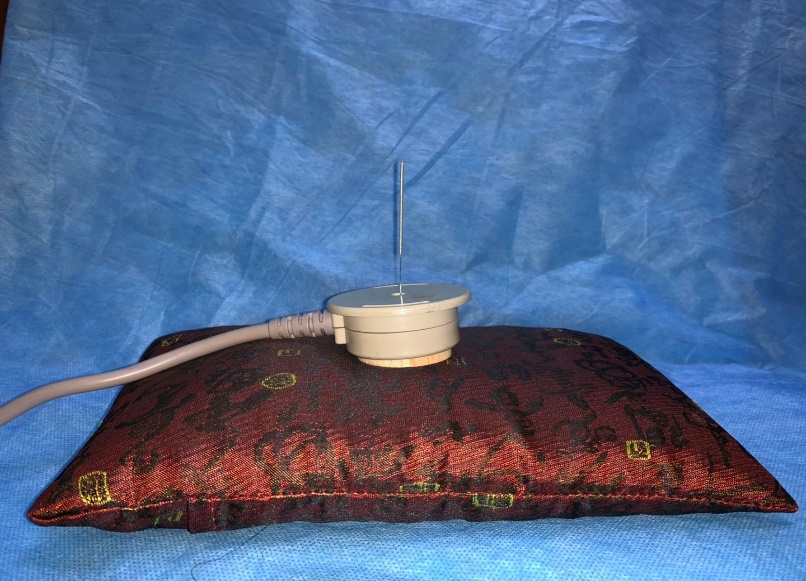

Supplement: Supplementary file 3 — Silver-needle warm acupuncture conducted Multi-Function Moxibustion Apparatus with specially designed heat pipe. (DOCX 722 kb) [file 13063_2019_3566_MOESM3_ESM.docx]
