# Supplementary material for: Evaluation of clinical efficacy of silver-needle warm acupuncture in treating adults with acute low back pain due to lumbosacral disc herniation: study protocol for a randomized controlled trial
Source: Trials. 2019 Jul 31;20:470. doi: 10.1186/s13063-019-3566-2 (PMC6668190; doi:10.1186/s13063-019-3566-2)
Supplement: Supplementary file 4 — Informed consent materials. (DOCX 17 kb) [file 13063_2019_3566_MOESM4_ESM.docx]

**Informed Consent Form**

Name of participant: Gender: Age: Registry No:

Dear participant:

This study is set to investigate the clinical efficacy of silver-needle warm acupuncture in treating lumbosacral disc herniation (LDH).

This is a clinical randomized controlled trial. If you agree to participate, you will be randomly assigned to an silver-needle warm acupuncture group or a conventional stainless filiform-needle warm acupuncture group. Therapies in both groups are relatively safe. If any unfavorable or unintended signs, symptoms, or diseases occur, you should report them to the doctors and they will take a positive response.

During the course of the study, you will need to: (1) complete the entire 9 sessions of treatment by the acupuncturist, (2) during the study, you will fill out some questionnaires and answer some questions to evaluate the efficacy of the treatment regimen for 4 times in total (before treatment, a week after the last treatment, 13 weeks and 25 weeks after the last treatment).

The participation in this study is entirely voluntary. Participants may be withdrawn at any time during the course of treatment without affecting the relationship between you and doctors. There is no loss in medical and economic aspects for you. You can also receive the effective treatment from doctors following routine medical procedures.

The research will strictly protect your privacy according to the principles of the Declaration of Helsinki. All information in this study will be kept confidential, and your private information will not appear in the research summary and published literature. This study has been ethically reviewed by the Ethics Committee of Shanghai Pudong Hospital of TCM on 2018 (pdzyyy-2018-21).

**Voluntary Subject Statement**:

I have learned about the requirements of the clinical study in detail and the potential adverse reactions associated with it. I voluntarily participate in this clinical study, receive treatment on time, and fill out the relevant health questionnaire in accordance with the requirements of the research program. If adverse reactions occur, I will report to the doctor promptly. At the same time I know that the doctor will give positive response for my possible adverse reactions. I am also entitled to withdraw from the study at any time for any reason. However, if there are no special circumstances, I will cooperate with the doctor to complete the clinical study. My participation and the personal data in the trial are confidential. I agree with my doctor, the relevant regulatory authorities, and the ethics committee to review my information as required.

I (signature) relative (signature) (Relationship)

Date: D M Y

**Doctor's declaration**

I have fully explained the requirements of the clinical study in detail and the potential adverse reactions to the above participant/relative, and answered their questions. To the best of my knowledge, the participant/relative has been informed adequately and has consented.

Doctor’s signature: Date: D M Y

*In the event of inconsistency or discrepancy between the Chinese version and the English version, the Chinese language version shall prevail.*
